# The *Haemophilus influenzae* HipBA toxin–antitoxin system adopts an unusual three-com­ponent regulatory mechanism

**DOI:** 10.1107/S205225252200687X

**Published:** 2022-07-29

**Authors:** Ji Sung Koo, Sung-Min Kang, Won-Min Jung, Do-Hee Kim, Bong-Jin Lee

**Affiliations:** a Research Institute of Pharmaceutical Sciences, College of Pharmacy, Seoul National University, Seoul 08826, Republic of Korea; b College of Pharmacy, Duksung Women’s University, Seoul 01369, Republic of Korea; c Jeju Research Institute of Pharmaceutical Sciences, College of Pharmacy, Jeju National University, Jeju 63243, Republic of Korea; d Interdisciplinary Graduate Program in Advanced Convergence Technology and Science, Jeju National University, Jeju 63243, Republic of Korea; Chinese Academy of Sciences, China

**Keywords:** HipBA, *Haemophilus influenzae*, toxin–antitoxin system, persister formation, X-ray crystallography

## Abstract

Type II toxin–antitoxin (TA) systems encode two proteins: a toxin that inhibits cell growth and an antitoxin that neutralizes the toxin by direct inter­molecular protein–protein inter­actions. The bacterial HipBA TA system is implicated in persister formation. The *Haemophilus influenzae* HipBA TA system consists of a HipB antitoxin and a HipA toxin, the latter of which is split into two fragments, and here we investigate this novel three-com­ponent regulatory HipBA system. Structural and functional analysis revealed that HipA^N^ corresponds to the N-ter­minal part of HipA from other bacteria and toxic HipA^C^ is inactivated by HipA^N^, not HipB. This study will be helpful in understanding the detailed regulatory mechanism of the HipBA^N+C^ system, as well as why it is constructed as a three-com­ponent system.

## Abstract 2

The crystal structure of HipA^N^, an unusual N-terminal com­ponent of the HipA toxin from *Haemophilus influenzae*, was determined at 2.70 Å resolution. A combined study on this unique three-com­ponent HipBA toxin–antitoxin system provides further insights into the regulatory mechanism of the HipBA toxin–antitoxin system.

## 1. Introduction

Prokaryotic toxin–antitoxin (TA) systems are encoded by small operons that are com­posed of two elements, a toxic subunit that causes cell-growth arrest and an antitoxic subunit that neutralizes the harmful effect of the toxin (Cheverton *et al.*, 2016 ▸). Depending on the mol­ecular mechanism by which the antitoxins neutralize their cognate toxins, TA systems have been classified into diverse types (Hayes, 2003 ▸). In the most abundant type II TA systems, the antitoxin is a protein that directly binds and neutralizes its cognate toxin through strong mol­ecular inter­actions (Hayes & Van Melderen, 2011 ▸). When freed from inhibition by antitoxins, the toxin acts on various targets to suppress cell growth or even induce cell death (Hall *et al.*, 2017 ▸). The toxin is relatively long-lived, whereas the antitoxin is degraded by cellular proteases due to its labile nature. Accordingly, the antitoxin should be constantly produced to regulate the toxin (Hõrak & Tamman, 2017 ▸). Type II antitoxins commonly contain a DNA-binding domain to bind at operators in the promoter region and repress the *TA* operon (Chan *et al.*, 2016 ▸). Therefore, as the amount of antitoxin is reduced, repression stops and the operon is transcribed to supply the antitoxin (Loris & Garcia-Pino, 2014 ▸). The cellular activity of a toxin is thus driven by the qu­antity of the cognate antitoxin (Hõrak & Tamman, 2017 ▸).

Type II TA systems are greatly abundant in nearly all free-living bacteria, raising debates about their biological functions (Pandey & Gerdes, 2005 ▸). Many studies point to additional functions of TA systems, including phage resistance, stress responses, biofilm formation and antibiotic persistence (Kędzierska & Hayes, 2016 ▸; Lobato-Márquez *et al.*, 2016 ▸; Díaz-Orejas *et al.*, 2017 ▸). Stress-induced activation of TA systems protects bacteria from adverse environmental conditions, such as antibiotic treatment, by causing persister formation (Radzikowski *et al.*, 2016 ▸). Anti­biotic persistence results in a transient slow-growing state of bacteria, which allows antibiotics to be tolerated (Harms *et al.*, 2016 ▸). This persister formation is a bet-hedging strategy in which the bacteria temporarily enters a nonreplicating state to gain stress tolerance against unfavourable environmental threats (Balaban *et al.*, 2004 ▸; Veening *et al.*, 2008 ▸; Lewis, 2010 ▸). Moreover, antibiotic persistence plays a crucial role in chronic infections and stimulates the evolution to antibiotic resistance (Levin-Reisman *et al.*, 2017 ▸).

The HipBA TA system was first identified in *Escherichia coli*, in which an increase in persister formation is associated with the *hipBA* operon (Moyed & Bertrand, 1983 ▸). As an abbreviation of ‘high incidence of persistence’, *hipB* (antitoxin gene) and *hipA* (toxin gene) constitute a type II TA module that encodes two proteins: HipB (antitoxin) and HipA (toxin) (Korch & Hill, 2006 ▸). HipA acts as a serine–threonine kinase, and the expression of HipA triggers considerable cell-growth arrest (Hanks *et al.*, 1988 ▸; Black *et al.*, 1991 ▸, 1994 ▸; Stancik *et al.*, 2018 ▸). On the other hand, HipB counteracts HipA by directly binding it and forming a stable protein com­plex (Korch & Hill, 2006 ▸). This HipBA protein com­plex binds to operators in the promoter region *via* the DNA-binding domain of HipB antitoxin and represses the transcription of *hipBA* (Schumacher *et al.*, 2015 ▸). Upon HipB degradation, free HipA induces multidrug tolerance, leading to the dormant state of cells (Veening *et al.*, 2008 ▸; Wood *et al.*, 2013 ▸).

This study elucidates the crystal structure of HI0666, which exhibits sequence similarity with the N-terminal part of HipA (HipA^N^). To date, the HipBA system has been structurally studied in only *E. coli* (Schumacher *et al.*, 2015 ▸) and *Shewanella oneidensis* (Wen *et al.*, 2014 ▸). Structural information obtained in this study shows that HI0666 is part of an unusual three-com­ponent TA system resembling the HipBA system. Compared with sequences of HipAs, whose structures were known previously, the HI0665 sequence exhibits similarity with the C-terminal sequence of HipA (HipA^C^) from other species. In the genome map, *hipA^C^* (encoding the C-terminus of HipA) is preceded by *hipA^N^* (encoding the N-terminus of HipA), and *hipA^N^* is preceded by *hipB* (encoding HipB). The results confirmed that HipA^N^ and HipA^C^ form a binary com­plex, and that HipB, HipA^N^ and HipA^C^ form a tertiary com­plex. HipA^C^-mediated growth inhibition was rescued by the expression of HipA^N^ but not by HipB. HipA^N^ functions as an untraditional antitoxin to HipA^C^, and HipB increases the ability of HipA^N^ to counteract HipA^C^. Taken together, these results outline a nontraditional three-com­ponent TA system, and a putative regulatory mechanism of the *H. influenzae* HipBA system is proposed.

## 2. Materials and methods

### 2.1. Gene cloning and transformation

*hi0666* (*hipA^N^*), a gene encoding HipA^N^ from *H. influenzae*, was amplified by polymerase chain reaction (PCR) using *H. influenzae* (KW20 strain, ATCC 51907) genomic DNA as a template. The oligonucleotide primers used for PCR are given in Table 1 ▸. After PCR amplification, *hipA^N^* was cloned into the pET28a vector (Novagen) using the restriction enzymes NdeI and XhoI. *hi0666.1* (*hipB*), a gene encoding HipB, was amplified in the same way and cloned into the pET21a vector (Novagen) using the same enzymes. To express HipA^N^ and HipA^C^ together, *hipA^N^* was first cloned into the pETDuet vector (Addgene), and *hi0665* (*hipA^C^*), a gene encoding HipA^C^, was subsequently introduced to follow *hipA^N^* in the pETDuet vector. For the cloning of *hipA^C^* into the pETDuet vector, the restriction enzymes BamHI and HindIII were used to digest PCR products and vector. A recombinant pET28a vector containing *hipA^C^* was also produced using the same approach. All recombinant plasmids were transformed into *E. coli* BL21(DE3) com­petent cells (Novagen).

### 2.2. Protein expression and purification

Transformed cells harbouring *hipA^N^* were grown to an optical density at 600 nm (OD_600_) of approximately 0.5 in LB medium containing 50 mg l^−1^ kanamycin at 37°C. At this point, expression was induced by adding 0.5 m*M* isopropyl β-d-1-thiogalactopyran­oside (IPTG) and cells were grown for an additional 4 h at the same temperature. Grown cells were harvested by centrifugation at 5600*g*. The harvested cells were resuspended in buffer A [50 m*M* Tris–HCl, pH 7.9, and 500 m*M* NaCl containing 10%(*v*/*v*) glycerol] and lysed by sonication. The cell lysate was centrifuged at 18 000*g* for 1 h, and the supernatant was applied to an affinity chromatography column of nickel–nitrilo­tri­acetic acid (Roche) equilibrated with buffer A. The trapped protein in the column was washed with buffer A containing 20 m*M* imidazole and eluted with buffer A containing 100–500 m*M* imidazole. Final purification of HipA^N^ was achieved by size-exclusion chromatography using a Superdex 200 (16/600PG) column (GE Healthcare) equilibrated with 20 m*M* HEPES, pH 7.5, and 500 m*M* NaCl. The purity of HipA^N^ was verified by SDS–PAGE.

### 2.3. Crystallization and diffraction data collection

The purified HipA^N^ was concentrated to 10 mg ml^−1^ using an Amicon Ultra Centrifugal Filter Unit (Millipore) for crystallization. Initial crystallization was performed with publicly available screening kits using a 384-well plate by the sitting-drop vapour-diffusion method. Each well of plates contained 0.5 µl of protein solution and 0.5 µl of reservoir solution. The plates were cultured at 4°C. The crystals used for data collection were grown in reservoirs with 2 *M* ammonium sulfate and 0.1 *M* Tris, pH 8.5. The X-ray diffraction data used for structure calculation were collected at 100 K using an ADSC Quantum 315r CCD detector at the 5C beamline of the Pohang Light Source, Republic of Korea. Collected raw data were processed and scaled with the *HKL2000* program suite (Otwinowski & Minor, 1997 ▸). Statistics for the data collection are described in Table 2 ▸.

### 2.4. Structure determination and refinement

To determine the structure of HipA^N^, *PhaserMR* in *Phenix* (Adams *et al.*, 2010 ▸) was employed using the Protein Data Bank (PDB) entry 2wiu (Evdokimov *et al.*, 2009 ▸) structure as a model. Iterative cycles of initial model building were performed by *Coot* (Emsley *et al.*, 2010 ▸), and further refinement was conducted using *REFMAC5* (Murshudov *et al.*, 1997 ▸). The HipA^N^ crystal structure belongs to the *P*4_1_2_1_2 space group at 2.70 Å. The coordinates and structure factors are deposited in the PDB under the accession code 7czo for HipA^N^, the N-terminal com­ponent of HipA toxin from *H. influenzae*. Detailed refinement statistics are available in Table 2 ▸.

### 2.5. Bioinformatics

Structure figures were generated using *PyMol* (*The PyMOL Mol­ecular Graphics System*, Version 1.8, Schrödinger, LLC). Superimpositions of structures were performed with the *SSM* (Krissinel & Henrick, 2004 ▸) option within *Coot*. The solvent-accessible areas of the protein surface were calculated by *PISA* (Krissinel & Henrick, 2007 ▸). Genome maps were utilized from the *KEGG* database (Kanehisa *et al.*, 2017 ▸). Sequence and structural similarity were searched using *DALI* (Holm & Rosenstrom, 2010 ▸). Structure-assisted alignment was carried out using *ESPript* (Robert & Gouet, 2014 ▸), with the help of *ClustalW* (Chenna *et al.*, 2003 ▸) for sequence alignments. The quality of the final structure was assessed at the wwPDB X-ray structure validation server (Berman *et al.*, 2003 ▸).

### 2.6. Copurification of HipA^N^, HipA^C^ and HipB

To determine whether HipB, HipA^N^ and HipA^C^ might form a protein com­plex, the pETDuet vector containing both *hipA^N^* and *hipA^C^*, and the pET21a vector containing *hipB* were used. For this study, a hexa­histidine tag was attached to the N-ter­minus of HipA^C^. These plasmids were cotransformed and bacterial cells were cultured in the same way as described above, with 50 mg l^−1^ streptomycin. Protein expression and affinity chromatography were also carried out in a similar manner to the above methods, but the three proteins were eluted carefully from being bound to the column with a gradient of 50–700 m*M* imidazole.

### 2.7. Analytical size-exclusion chromatography

The purified protein mixture of HipB, HipA^N^ and HipA^C^ was subjected to size-exclusion chromatography under the same conditions as the above mixture. A standard curve was obtained using standards from gel-filtration calibration kits (GE Healthcare), including aldolase (158 kDa), conalbumin (75 kDa) and ovalbumin (43 kDa), and the curve was com­pared with the peak position obtained from the protein mixture.

### 2.8. Cell-growth assay

To validate the three-com­ponent regulatory mechanism of the *H. influenzae* HipBA system, a cell-growth assay was performed using the following plasmids resistant to different antibiotics: pET21a containing *hipB*, pETDuet containing only *hipA^N^* and pET28a containing *hipA^C^*. For this assay, all plasmids were transformed into *E. coli* strain BL21(DE3). Single colonies from transformed cells grown in M9 medium plates containing 0.1% glucose were further grown overnight. These overnight cultures were then diluted to an OD_600_ of 0.1. The diluted cells were freshly grown until the OD_600_ reached 0.5; 0.5 m*M* IPTG was then added to the culture medium to induce protein expression. The cells were incubated at 37°C and monitored at 1 h inter­vals.

## 3. Results and discussion

### 3.1. Overall structure of *H. influenzae* HipA^N^ and the *hipBA* genome map

**Figure 1:**
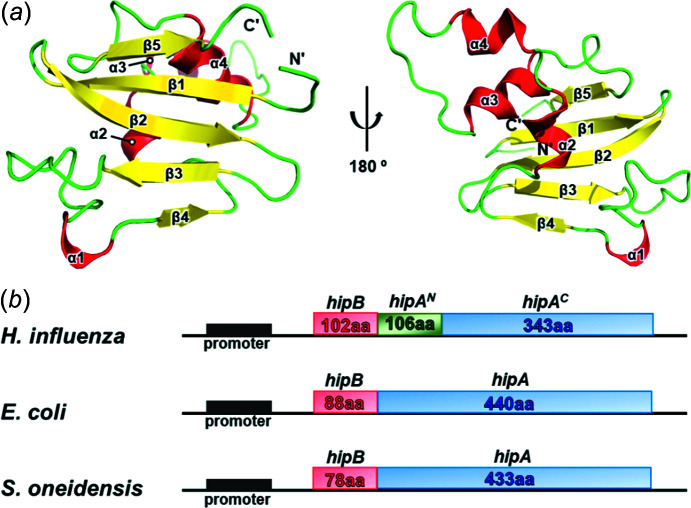
The overall structure of the *H. influenzae* HipA^N^ and *hipBA* genome maps. (*a*) 180° rotated views of *H. influenzae* HipA^N^. α-Helices, β-strands and loops are coloured red, yellow and green, respectively. (*b*) Schematic diagram showing a com­parison of the *hipBA* operons of *H. influenzae*, *E. coli* and *S. oneidensis*.

There are four α-helices and five β-strands arranged as a β-barrel in the structure of *H. influenzae* HipA^N^ [Fig. 1 ▸(*a*)]. Among those β-strands, five β-strands (β1–β5) form a β-sheet antiparallel to each other and these antiparallel β-sheets are flanked by four α-helices. The total solvent-accessible surface area of the monomeric structure is 7119 Å^2^.

In the reported structures of HipBA systems, we discovered an additional locus in the genome map of *H. influenzae* immediately upstream from *hipA^C^*. This locus is called *hipA^N^* in this article and encodes a 106 aa protein corresponding to the N-terminal part of HipA from *E. coli* and *S. oneidensis* [Fig. 1 ▸(*b*)]. In all these cases, *hipB* was located upstream of *hipA* and these putative HipB homologs might thus autoregulate the *hipBA^N+C^* operons. In summary, the *hipBA* operon contains two genes, *hipB* and *hipA*, while the *hipBA* operon from *H. influenzae* contains three genes, *hipB*, *hipA^N^* and *hipA^C^*.

### 3.2. Comparative structural analysis of the HipBA system

**Figure 2:**
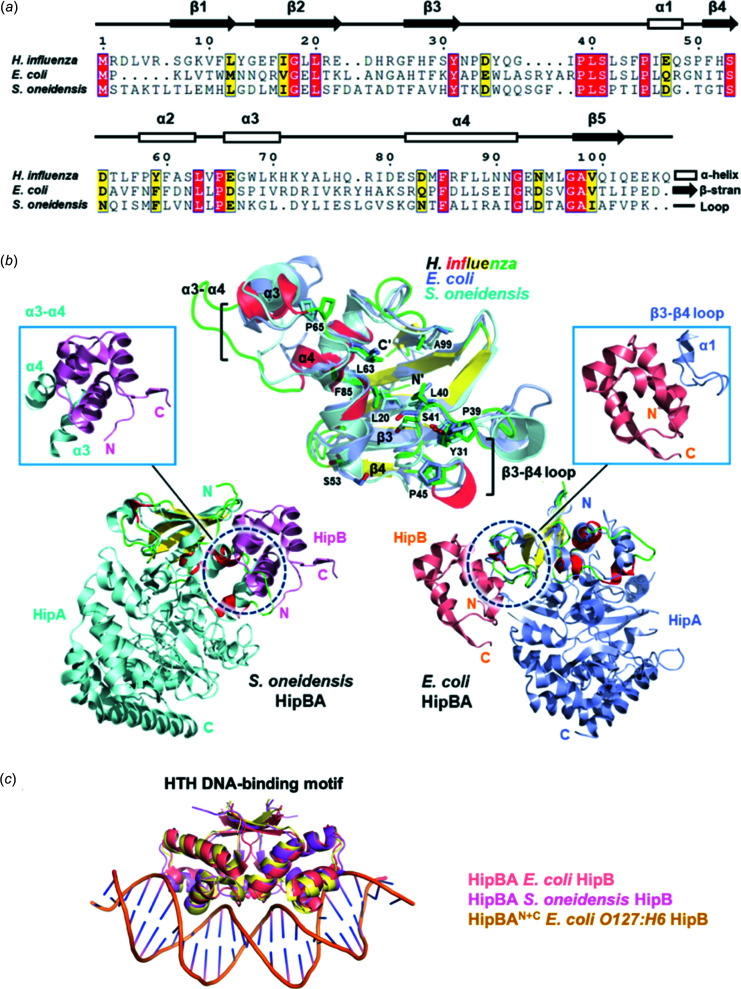
Comparative structural analysis of HipBA systems. (*a*) Structure-guided sequence alignment of *H. influenzae* HipA^N^ with the N-terminal parts of other HipAs. The secondary structural organization of *H. influenzae* HipA^N^ is displayed on the alignment. Conserved residues are highlighted in red and yellow. (*b*) Structural com­parison of *H. influenzae* HipA^N^ and HipBA systems from other organisms. Residues showing high conservation are marked in the overlay. HipBA binding inter­faces of *E. coli* and *S. oneidensis* are identified in circles and enlarged in squares. (*c*) Structural com­parison of HipB antitoxins from *E. coli O127:H6* (PDB code: 7ab3) and *S. oneidensis* (PDB code: 4pu3) using *E. coli* HipB antitoxin bound to the DNA (PDB code: 5k98) structure as model.

To date, structures of HipA have been reported from two species: *E. coli* (PDB code 5k98; *Z* score of 9.2, r.m.s. deviation of 2.4 Å, sequence identity of 24%) (Schumacher *et al.*, 2015 ▸) and *S. oneidensis* (PDB code 4pu3; *Z* score of 9.9, r.m.s. deviation of 2.7 Å, sequence identity of 23%) (Wen *et al.*, 2014 ▸) [Fig. 2 ▸(*a*)]. To com­pare the structural characteristics of *H. influenzae* HipA^N^ and its homologs, a com­parative analysis was performed with these two reported structures. *H. influenzae* HipA^N^ superimposed well with the N-terminal parts of the HipA proteins from *E. coli* and *S. oneidensis* [Fig. 2 ▸(*b*)]. In addition, *H. influenzae* HipB showed a 48% sequence similarity to HipB of the *E. coli O127:H6* tripartite HipBA system (PDB code: 7ab3), which in turn showed high structural homology with *E. coli* HipB (PDB code: 5k98, *Z* score of 12.0, r.m.s. deviation of 1.2 Å) (Schumacher *et al.*, 2015 ▸) and *S.oneidensis* HipB (PDB code: 4pu3, *Z* score of 9.7, r.m.s. deviation of 1.8 Å) (Wen *et al.*, 2014 ▸) [Fig. 2 ▸(*c*)]. Regardless of the binary or tertiary nature of the HipBA systems, dimeric HipB antitoxins were highly conserved in their DNA-binding HTH motif [Fig. 2 ▸(*c*)], which also indicated their conserved role as transcriptional regulators. Furthermore, although the HipA toxins had similar folds, the N-terminal of HipA bound HipB antitoxins differently with respect to *E. coli* (loop between β3 and β4, containing α1) and *S. oneidensis* (α3–α4) proteins. This may suggest that the toxin neutralization mechanism of the HipBA system might be different among its homologues.

### 3.3. Complex formation of HipB, HipA^N^ and HipA^C^

**Figure 3:**
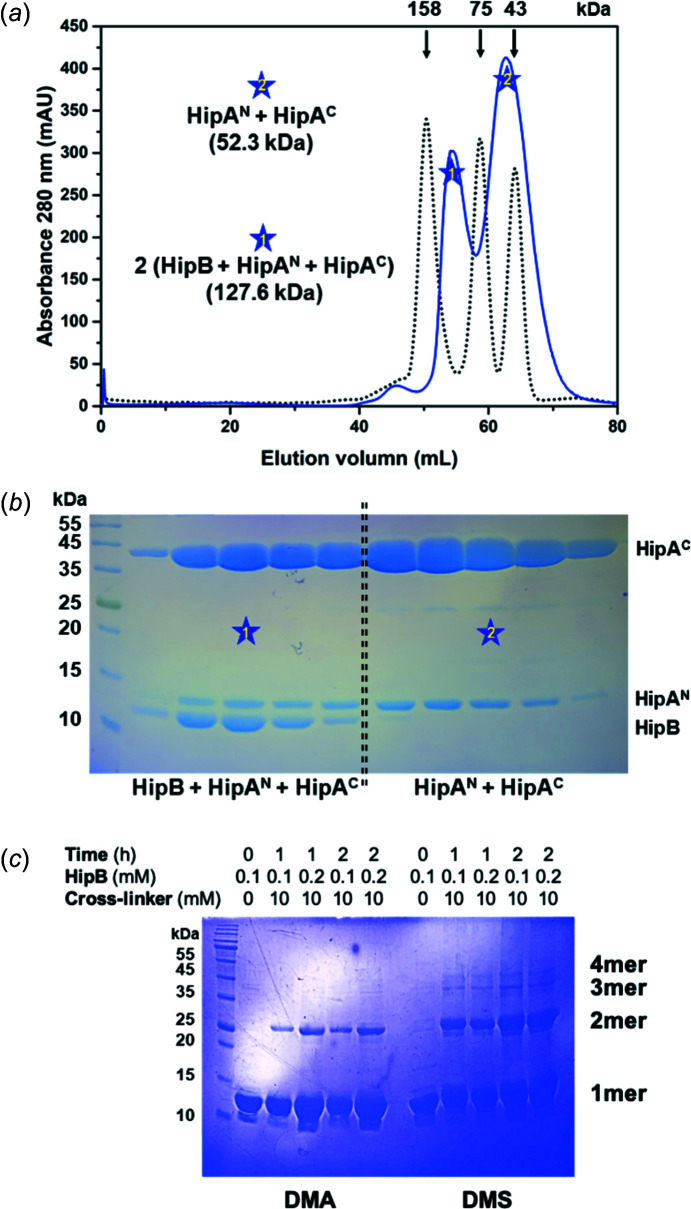
Complex formation of HipB, HipA^N^ and HipA^C^. (*a*) Purification of tertiary mixtures using size exclusion chromatography. Elution locations of standard proteins with known mass are shown with vertical arrows. The two major peaks of the protein mixture are emphasized with star symbols. (*b*) SDS–PAGE analysis of size exclusion chromatography fractions. Star symbols indicate the lanes to which the starred peaks belong. (*c*) SDS–PAGE analysis of chemical crosslinking of *H. influenzae* HipB using DMA and DMS crosslinkers. Experimental conditions are indicated above and the corresponding multimer form is labelled on the right side of the gel.

To show that HipB, HipA^N^ and HipA^C^ from *H. influenzae* form a com­plex similar to those of other type II TA systems, analytical gel filtration was conducted [Fig. 3 ▸(*a*)], and the eluted fractions were analyzed by SDS–PAGE [Fig. 3 ▸(*b*)]. His-tagged HipA^C^ was pulled down with both HipA^N^ and HipB. Size-exclusion chromatography further confirmed that two forms of com­plex [(HipB + HipA^N^ + HipA^C^) and (HipA^N^ + HipA^C^)] are monodispersed in solution. The second peak eluted between the 75 and 43 kDa mol­ecular weight standards, which corresponded to the heterodimeric HipA^N+C^ com­plex (∼52.3 kDa). This was consistent with the relative masses of HipA toxin structures in the binary *E. coli* HipBA systems (Schumacher *et al.*, 2015 ▸). In addition, chemical crosslinking of *H. influenzae* HipB showed the predominant multimeric form of HipB as the dimer [Fig. 3 ▸(*c*)], as the other HipB antitoxins and thus the results of the first peak which eluted between the 158 and 75 kDa mol­ecular weight standards were presumed to be the hexa­meric com­plex consisting of one HipB dimer bound to two HipA^N+C^ heterodimers. As predicted, the recently deposited structure of the tertiary HipBA com­plex (PDB code: 7ab3) in *E. coli O127:H6* revealed a hexa­meric assembly in which the HipB dimer was bound to two HipA^N+C^ hetero­dimers (Baerentsen *et al.*, 2022 ▸). Indeed, the expected mol­ecular weights of the copurified three proteins suggested that *H. influenzae* (HipB + HipA^N^ + HipA^C^) forms a hetero­hexamer and (HipA^N^ + HipA^C^) forms a heterodimer in solution.

### 3.4. Three-com­ponent regulatory mechanism of the *H. influenzae* HipBA system

**Figure 4:**
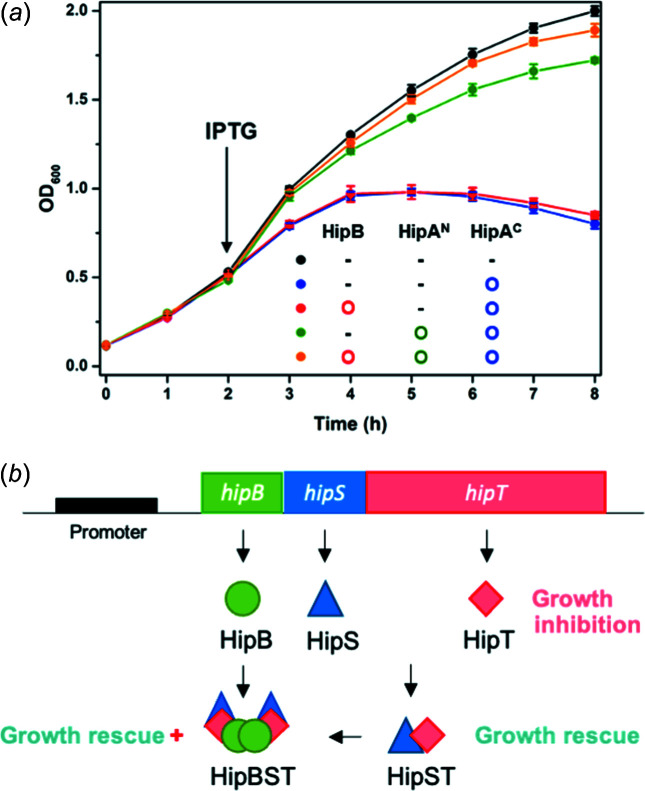
Three-com­ponent regulatory mechanism of the *H. influenzae* HipBA system. (*a*) Cell-growth analysis showing the effects of expression of different proteins. Each curve represents the growth of cells with a different combination of proteins, as presented within the graph. The data show the average values obtained by triplicate assays; the standard deviations are indicated by error bars. (*b*) Schematic overview of the presumptive regulatory mechanism according to the inter­actions between the com­ponents of the *H. influenzae* HipBA system.

We validated the com­ponents constituting the *H. influenzae* HipBA system and their regulatory mechanism through a cell-growth assay [Fig. 4 ▸(*a*)]. Expression of HipA^C^ resulted in strong inhibition of cell growth, indicating that HipA^C^ functions as a toxin. Surprisingly, the growth was not rescued by the traditional HipB antitoxin but was rescued upon the expression of HipA^N^, suggesting that HipA^N^ functions as the antitoxin. In addition, co-induction of HipB and HipA^N^ provided a slightly advantageous growth rescue com­pared to HipA^N^ alone, giving rise to the possibility that HipB may augment the antitoxin activity of HipA^N^ to quickly restore cell growth [Fig. 4 ▸(*b*)]. The co-induction of HipB and HipA^N^ in the *E. coli O127:H6* HipBA tripartite TA system also showed improved growth over induction of HipA^N^ alone (Vang Nielsen *et al.*, 2019 ▸), further supporting the augmentative role of HipB along with the HipA^N^ com­ponent to further inhibit the toxic activity of HipA^C^ toxins.

## 4. Conclusions

Of the three proteins constituting the *H. influenzae* HipBA^N+C^ system, the function of HipA^N^ as the third com­ponent is quite striking. We found that HipA^N^ counteracts the toxin HipA^C^, while the conserved HipB does not have an antitoxic effect. However, HipB is proposed to strengthen the activity of the HipA^N^-mediated neutralization of HipA^C^, supporting its augmentative role. For example, HipB might intensify the efficacy of HipA^N^ by stabilizing its inter­action with HipA^C^. This hypothesis is consistent with the com­plex formation of HipB, HipA^N^ and HipA^C^. In addition, *hipA* might have been split into two fragments during evolution, but the reason for this is unknown.

In conclusion, our work here reveals a novel type of three-com­ponent TA module with unknown regulator properties that is important and exciting to study.

## Supplementary Material

PDB reference: N-terminal domain of HipA toxin, 7czo

## Figures and Tables

**Table 1:** Primers designed for cloning the genes encoding HipA^N^, HipA^C^ and HipB

Proteins (vector)		Primer sequence (5′→3′)
HipA^N^ (pET28a)	Forward	GGAATTC CATATG ATG CGC GAT TTA GTC CG
	Reverse	CCG CTCGAG TTA TTG TTT TTC TTC CTG AAT TTG C
HipB (pET21a)	Forward	GGAATTC CATATG ATG GAC AAT CTT AGT GCA C
	Reverse	CCG CTCGAG TTA AAT CGC GCA TAG TGA AAC
HipA^N^ (pETDuet)	Forward	GGAATTC CATATG ATG CGC GAT TTA GTC
	Reverse	CCG CTCGAG TTA TTG TTT TTC TTC CTG AAT TTG
HipA^C^ (pETDuet)	Forward	CGC GGATCC G ATG AAT TTT TGT CGT ATT TTA
	Reverse	CCC AAGCTT TTA TAG TTC AGG TTC ATT TAA TAG
HipA^C^ (pET28a)	Forward	CGC GGATCC G ATG AAT TTT TGT CGT ATT TTA TTA AAG CCA
	Reverse	CCC AAGCTT TTA TAG TTC AGG TTC ATT TAA TAG GTT AAG CA

**Table 2:** Statistics for data collection and model refinement Values in parentheses refer to the highest-resolution shell.

	HipA^N^ (PDB code: 7czo)
Data collection	
X-ray wavelength (Å)	0.9795
Space group	*P*4_1_2_1_2
Unit-cell length (*a*, *b*, *c*, Å)	66.25, 66.25, 103.62
Unit-cell angle (α, β, γ, °)	90.00, 90.00, 90.00
Resolution range (Å)	50.00–2.70 (2.75–2.70)
Total/unique reflections	94,246/6,880 (345)
Completeness (%)	99.9 (100.0)
CC_1/2_ †	0.998 (0.973)
*I*/σ_*I*_	27.9 (3.2)
*R*_merge_ ‡	0.096 (0.766)
	
Model refinement	
*R*_work_/*R*_free_ §	0.242/0.274
No./average *B* factor (Å^2^)	
Protein atoms	882/41.43
Water oxygen atoms	30/45.88
R.m.s. deviation from ideal geometry	
Bond lengths (Å)/bond angles (°)	0.011/1.473
Ramachandran plot (%)	
Most favorable	99.42
Allowed	0.58
Disallowed	0.00

† CC_1/2_ is described in Karplus & Diederichs (2012 ▸).

‡ *R*_merge_ = 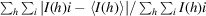 , where *I*(*h*) is the intensity of reflection *h*, 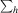 is the sum over all reflections and 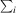 is the sum over *i* measurements of reflection *h*.

§ *R* = 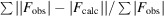 , where *R*_free_ is calculated for a randomly chosen 5% of reflections that were not used for structure refinement and *R*_work_ is calculated for the remaining reflections.
